# Direct In Vitro Comparison of the Anti-Leishmanial Activity of Different Olive Oil Total Polyphenolic Fractions and Assessment of Their Combined Effects with Miltefosine

**DOI:** 10.3390/molecules27196176

**Published:** 2022-09-21

**Authors:** Georgia Gogou, Olga S. Koutsoni, Panagiotis Stathopoulos, Leandros A. Skaltsounis, Maria Halabalaki, Eleni Dotsika

**Affiliations:** 1 Laboratory of Cellular Immunology, Department of Microbiology, Hellenic Pasteur Institute, 11521 Athens, Greece; 2 Division of Pharmacognosy and Natural Product Chemistry, Department of Pharmacy, National and Kapodistrian University of Athens, 15784 Athens, Greece

**Keywords:** leishmaniasis, natural products, total phenolic fraction, promastigotes, intracellular amastigotes, isobologram, combination therapy

## Abstract

The bioactive compounds present in the edible products of the olive tree have been extensively studied and their favorable effects on various disease risk factors have been demonstrated. The aim of this study was to perform a comparative analysis of the anti-leishmanial effects of total phenolic fractions (TPFs) derived from extra virgin olive oil with different phenolic contents and diverse quantitative patterns. Moreover, the present study investigated their association with miltefosine, a standard anti-leishmanial drug, against both extracellular promastigotes and intracellular amastigotes of a viscerotropic and a dermotropic *Leishmania* strain. The chemical compositions of TPFs were determined by high performance liquid chromatography with diode array detection (HPLC-DAD). Analysis of parasite growth kinetics, reactive oxygen species production and apoptotic events were determined by microscopy and flow cytometry. Our results revealed that the presence of oleacein (OLEA) and oleocanthal (OLEO) secoiridoids enhances the anti-leishmanial effect of TPF. The association between TPFs and miltefosine was suggested as being additive in *Leishmania infantum* and *Leishmania major* promastigotes, and as antagonistic in intracellular amastigotes, as was evaluated with the modified isobologram method. The obtained data verified that TPFs are bioactive dietary extracts with a strong anti-leishmanial activity and highlighted that fractions that are richer in OLEA and OLEO phenolic compounds possess stronger inhibitory effects against parasites. This study may contribute to improving the therapeutic approaches against leishmaniasis.

## 1. Introduction

Leishmaniasis is a vector-borne infectious disease, caused by several protozoan parasites of the genus *Leishmania* [1]. It has a broad spectrum of clinical manifestations, and it is mainly classified into three forms: visceral, cutaneous, and mucocutaneous. Visceral leishmaniasis (VL) is the most severe and systemic form, which can be lethal if left untreated, while cutaneous leishmaniasis (CL) is the most prevalent form and is usually limited to an ulcer that self-heals, but can also lead to disfiguring scars and disabilities [2]. It is estimated that 50,000 to 90,000 new cases of VL and 600,000 to 1,000,000 new cases of CL occur worldwide annually [3].

Since a vaccine is currently unavailable, chemotherapy is the only treatment option for leishmaniasis [4,5]. Among the various anti-leishmanial treatments, pentavalent antimonials were the first-line drugs applied against leishmaniasis over several decades P [6,7]. Nowadays, the available monotherapies and combination treatments include various chemotherapeutic resources, such as amphotericin B, pentamidine, paromomycin, various azoles, and miltefosine [8,9,10]. Nevertheless, all the existing drugs present several drawbacks, such as a high cost, toxicity, and the emergence of resistance. Thus, the treatment of leishmaniasis remains a challenge and the search for new active compounds is an urgent need.

Natural products, as either herbal extracts or plant-derived compounds, comprise valuable sources of drugs and many of them have been tested as anti-parasitic and anti-leishmanial drugs [5,8,11]. In addition, combination therapies might be an alternative approach by reducing the overall dose of drugs and the treatment duration [12,13,14]. The olive tree *Olea europaea* L. (Oleacea), which extensively grows in the Mediterranean region, has long been known to contain a wealth of biologically active compounds with health-promoting potential [15,16]. Extra virgin olive oil (EVOO) is mainly composed of lipophilic components that are rich in monounsaturated fatty acids (MUFA). It also consists of the polar fraction (total phenolic fraction—TPF), which contains diverse chemical classes of polyphenols, such as phenyl alcohols, phenolic acids, secoiridoids, flavonoids, and lignans, which are associated with health-beneficial properties, such as antioxidant, anti-inflammatory and anti-cancer properties [17,18,19,20]. According to the EU Health Claim Labeling Regulation, olive oil polyphenols are therefore listed among food substances with attributed health claims (432/2012) [21,22]. Our group has showcased the anti-leishmanial properties of olive oil phenolic compounds by demonstrating that pure oleuropein and oleocanthal (OLEO) exert anti-leishmanial properties in vitro and promote in vivo efficacy in murine experimental models of VL and CL [23,24,25,26]. In addition, we have reported that TPF exhibits chemotherapeutic anti-leishmanial activity in vitro and in vivo [27,28].

This study aimed (i) to perform an in vitro comparative analysis of the anti-leishmanial properties of two distinct TPFs with completely different phenolic contents with respect to the two main chemical classes of polyphenols, secoiridoids (oleacein—OLEA, and oleocanthal—OLEO) and phenyl alcohols (hydroxytyrosol—HT, and tyrosol—T), against a viscerotropic and a dermotropic *Leishmania* strain, and (ii) to determine their combinatorial association with the miltefosine standard anti-leishmanial drug (hexadecylphosphocholine/HePC), on both parasite developmental forms, the extracellular promastigotes and the intracellular amastigotes. As treatment of the disease is challenging, the present study may contribute to the development of an effective treatment scheme against leishmaniasis based on bioactive components that are naturally presented in the diet.

## 2. Results

### 2.1. Chemical Composition of Total Phenolic Fraction 1 (TPF1) and Total Phenolic Fraction 2 (TPF2)

**Figure 1:**
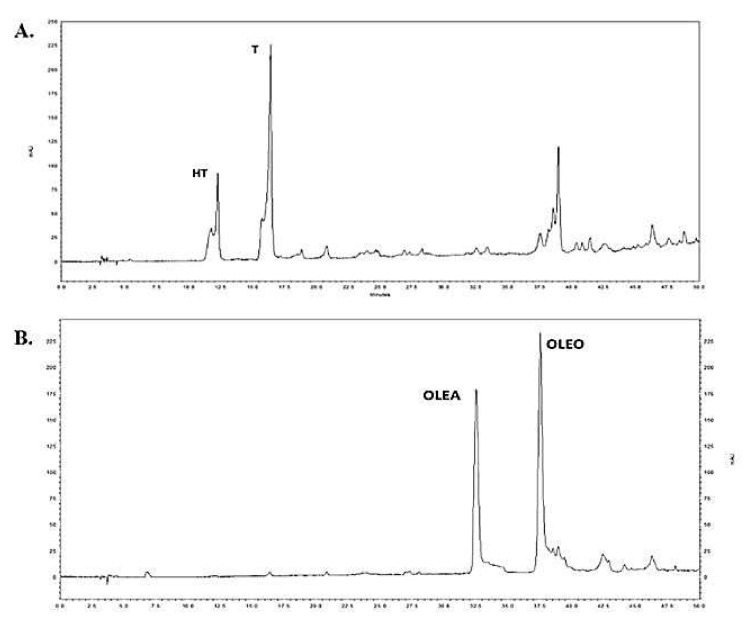
HPLC-DAD chromatograms obtained from the analysis of TPF1 (**A**) and TPF2 (**B**) extracts, applying the IOC proposed method. Hydroxytyrosol (HT), tyrosol (T), oleacein (OLEA), and oleocanthal (OLEO) peaks are indicated.

The chemical analysis performed on the EVOO-1 and EVOO-2 samples revealed that TPF1 was rich in HT and T while TPF2 was enriched in OLEA and OLEO (Figure 1). Specifically, the content of HT and T in the EVOO-1 extract was 7.02 and 42.07 mg/g of TPF, while the concentration levels of OLEA and OLEO were below the detection limit of the HPLC-DAD method. In the EVOO-2 extract, the content of HT and T was detected approximately at the same levels as TPF1 (5.01 and 12.03 mg per g of extract), while the concentrations of OLEA and OLEO were at higher levels, 144.12 and 301.24 mg/g, respectively (Table 1).

### 2.2. Anti-Promastigote Activity Evaluation (IC_50_) of TPF1 and TPF2 against Leishmania spp.

**Figure 2:**
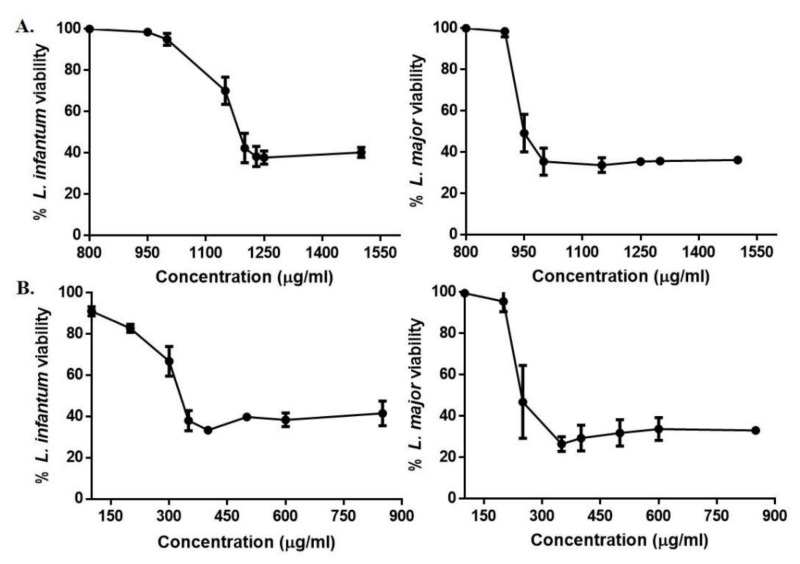
Anti-promastigote effect of TPF1 and TPF2 against *L. infantum* and *L. major* promastigotes. Log-phase promastigotes were treated with TPF1 (**A**) and TPF2 (**B**) at various increasing concentrations for 72 h and their viability was determined using the resazurin cell viability assay. OD was determined with a microplate spectrophotometer at 570 nm (reference filter 630 nm). Data are presented as mean values ± SD of three independent experiments.

The biological effects of TPF1 and TPF2 against promastigote forms of two different *Leishmania* species, were evaluated at various increasing concentrations, ranging from 800 to 1500 µg/mL and 100 to 850 µg/mL, respectively. After 72 h of incubation, both TPF1 and TPF2 demonstrated inhibitory effects on the viability of *L. infantum* and *L. major* promastigotes in a dose-dependent manner in comparison with untreated parasites (Figure 2). Substantial inhibition was observed at higher concentrations and the IC_50_ values of TPF1 and TPF2 against *L. infantum* promastigotes were determined at 1186.48 ± 45.82 and 322.58 ± 18.87 µg/mL, respectively, while the relevant IC_50_ values against *L. major* promastigotes were determined at 976.03 ± 21.56 µg/mL for TPF1 and 252.58 ± 30.16 µg/mL for TPF2 (Table 2). Thus, both *L. infantum* and *L. major* promastigotes were more susceptible to TPF2. 

### 2.3. Anti-Amastigote Activity Evaluation (IC_50_), Cytotoxicity (CC_50_) and Selectivity Index Calculation (SI)

Since *Leishmania* spp. have two clearly defined developmental forms: promastigote forms that colonize insect vectors and amastigote forms that multiply primarily within mammalian macrophages [29,30], we therefore tested the activity of TPF1 and TPF2 on intracellular *L. infantum* and *L. major* amastigotes. After the treatment of *Leishmania*-infected macrophages with different concentrations of TPF1 and TPF2, we found a significant inhibition of intracellular amastigotes. The anti-amastigote activity of TPF1 and TPF2 is summarized in Table 2 and the IC_50_ values were determined at 207.02 ± 6.57 and 104.98 ± 10.02 for *L. infantum* and 142.3 ± 28.24 and 76.07 ± 14.5 for *L. major*, respectively, indicating that both *L. infantum* and *L. major* amastigotes were also more susceptible to TPF2, as was observed with the promastigote forms. We also determined through the resazurin reduction assay the toxicity of TPF1 and TPF2 on J774A.1 macrophages and we further compared this toxicity with their anti-amastigote activity by calculating the selectivity index (SI = CC_50_ for J774A.1 cells/IC_50_ for amastigotes) (Table 2). Our results demonstrated that both TPF1 and TPF2 were selective against *Leishmania* spp. 

### 2.4. Effect of TPF1 and TPF2 on Growth Kinetics of Leishmania spp. Promastigotes

**Figure 3:**
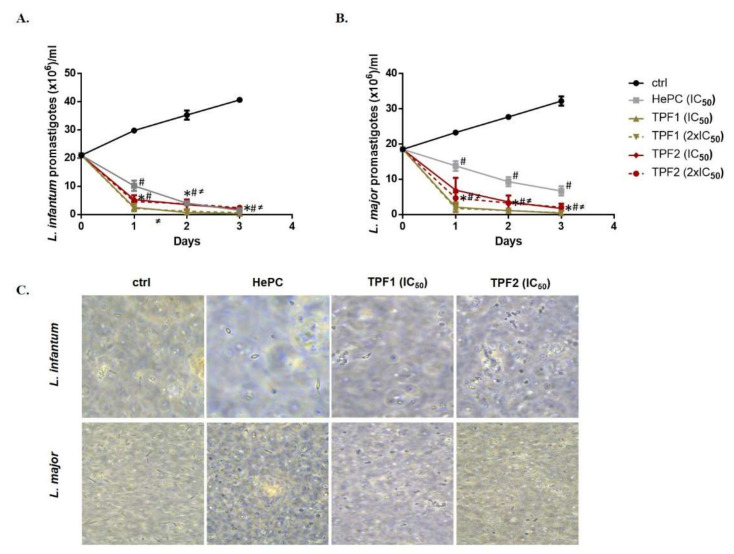
Effect of TPF1 and TPF2 on *Leishmania* spp. promastigotes growth kinetics. Log-phase *L. infantum* (**A**) and *L. major* (**B**) promastigotes were treated with TPF1 and TPF2 at IC_50_ and 2 × IC_50_ doses. The parasite growth and multiplication were determined by differential counting of dead and live promastigotes at 24 h intervals over a period of 72 h. HePC (IC_50_)-treated and untreated parasites were used as positive and negative control groups, respectively. Data are expressed as mean value ± SD of three independent experiments. Symbols of * and # indicate significant differences compared to positive and negative control groups, respectively. Differences between TPF1- and TPF2-treated parasites are indicated with ≠. (**C**) Determination of the promastigote morphology by phase-contrast microscopy (40× magnification).

The growth kinetics of log-phase *L. infantum* and *L. major* promastigotes were performed in vitro after their treatment with IC_50_ and 2 × IC_50_ concentrations of TPF1 and TPF2, independently. The results were obtained by counting daily the promastigote number of each *Leishmania* strain over a 72 h treatment period. The growth curves are shown in Figure 3. With initial population densities of 20 × 10^6^ parasites/mL, both *L. infantum* and *L. major* untreated parasites exhibited an exponential growth, while treated parasites with TPF1 and TPF2 or the standard drug (HePC), had analogous growth patterns, indicating a significant reduction evident from the first 24 h of treatment. The effect of TPF1 and TPF2 in the culture growth of both *Leishmania* strains was more potent compared to HePC (*p* ≤ 0.05). Specifically, *L. infantum* and *L. major* promastigotes demonstrated an 88.1% and an 88.6% decrease when treated with TPF1 at an IC_50_ concentration and an 89.8% and a 90.8% decrease when they were treated with TPF1 at 2 × IC_50_ concentration, respectively, after 24 h of treatment. The relative decrease in HePC-treated parasites was 51.7% and 25.4%, respectively. Additionally, *L. infantum* and *L. major* promastigotes demonstrated a 75% and a 62.7% decrease when they were treated with TPF2 at an IC_50_ concentration and a 78.3% and a 74.6% decrease when they were treated with 2 × IC_50_ concentration, respectively. In order to illustrate the anti-promastigote effects of TPF1 and TPF2, phase-contrast micrographs of *L. infantum* and *L. major* treated promastigotes were taken at 72 h of treatment (Figure 3C).

### 2.5. TPF1 and TPF2 Induce ROS Generation in Leishmania spp. Promastigote

**Figure 4:**
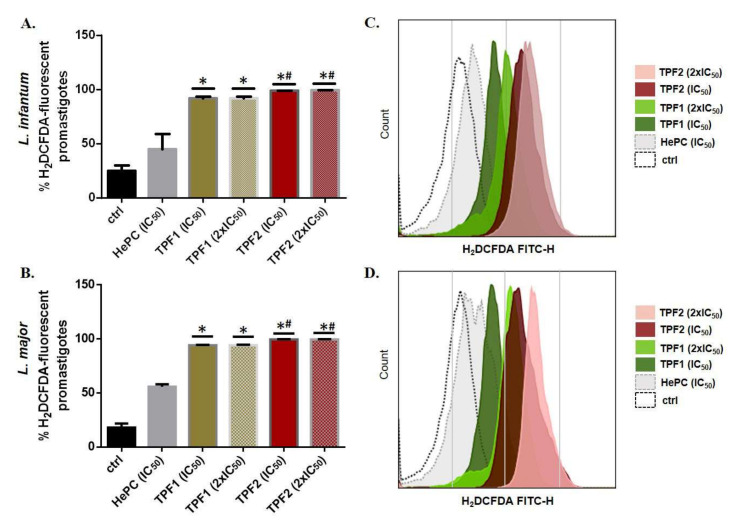
Intracellular ROS levels in TPF1- and TPF2-treated *Leishmania* spp. promastigotes. Log-phase *L. infantum* and *L. major* promastigotes were treated with TPF1 and TPF2 at IC_50_ and 2 × IC_50_ doses for 72 h. HePC (IC_50_)-treated and untreated parasites were used as positive and negative control groups, respectively. ROS levels were quantified using H_2_DCFDA and flow cytometry. Data are presented as mean values of H_2_DCFDA-fluorescent *L. infantum* (**A**) and *L. major* (**B**) promastigotes (±SD) in bar diagrams, and as single parameter histograms (FITC-H) for *L. infantum* (**C**) and *L. major* (**D**). The symbol of * indicates significant differences compared to the positive control group. Differences between TPF1- and TPF2-treated parasites are indicated with #.

Staining with H_2_DCFDA was performed to evaluate TPF1- and TPF2-induced ROS generation in *L. infantum* and *L. major* promastigotes. ROS generation was remarkably enhanced to 91.8% and 98.9% in *L. infantum* promastigotes treated with TPF1 and TPF2 for 72 h, at concentrations corresponding to their IC_50_ values (*p* = 0.021 and 0.020), compared to the untreated parasites (Figure 4A). Similarly, treatment with TPF1 and TPF2 also generated ROS production in *L. major* promastigotes. We observed 94.2% and 99.5% H_2_DCFDA-fluorescent parasites upon treatment with IC_50_ concentrations, respectively, while the relevant percentage in the control parasites was 18.1% (*p* = 0.014 and 0.009) (Figure 4B). In addition, we also observed a significant shift of fluorescence in the right direction at IC_50_ and 2 × IC_50_ concentrations for both TPF1 and TPF2 (Figure 4C,D). Our results indicated that TPF2 was more effective compared to TPF1 in both *Leishmania* strains (*p* ≤ 0.02). *Leishmania* spp. promastigotes were also treated with hydrogen peroxide (H_2_O_2_), serving as the experimental positive control and it induced ROS formation to 55.6% and 52.6% in *L. infantum* and *L. major* promastigotes, respectively (data not shown).

### 2.6. TPF1 and TPF2 Induce Phosphatidylserine Externalization and Cause Loss of Cell Membrane Integrity in Leishmania spp. Promastigotes

**Figure 5:**
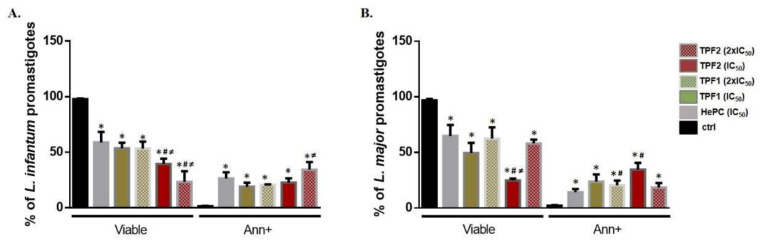
Changes in phosphatidylserine exposure in TPF1- and TPF2-treated *Leishmania* spp. promastigotes. Log-phase *L. infantum* (**A**) and *L. major* (**B**) promastigotes were either left untreated (negative control) or were treated with IC_50_ and 2 × IC_50_ doses of TPF1, TPF2 and HePC (positive control) for 72 h. Parasites were labelled with annexin V-FITC and PI. Data are presented as mean values (± SD) of viable (annexin V-/PI- parasites) and annexin V+ parasites in bar diagrams that are representative of three independent experiments. The symbols of * and # indicate significant differences compared to the negative and positive control groups, respectively. Significant differences between TPF1- and TPF2-treated parasites are indicated with ≠.

**Figure 6:**
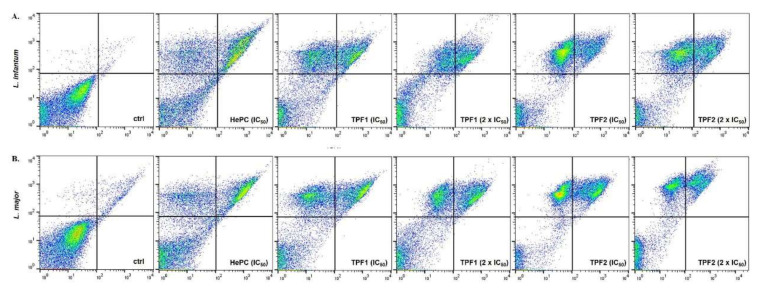
Changes in phosphatidylserine exposure and plasma membrane integrity in TPF1- and TPF2-treated *L. infantum* and *L. major* promastigotes. Log-phase *L. infantum* (**A**) and *L. major* (**B**) promastigotes were either left untreated (negative control) or were treated with IC_50_ and 2 × IC_50_ doses of TPF1, TPF2 and HePC (positive control) for 72 h. Parasites were labelled with annexin V-FITC and PI that discriminate between early (annexin V+/PI-) and late (annexin V+/PI+) apoptotic, necrotic (annexin V-/PI+) and live (annexin V-/PI-) parasites. Phosphatidylserine exposure and cell membrane permeability were quantified by flow cytometry and data are presented as flow cytometric dot plots with respective quadrants, representative of one experiment.

Staining with annexin V-FITC and propidium iodide (PI) was performed to evaluate TPF1- and TPF2-induced phosphatidylserine externalization and cell membrane integrity in *L. infantum* and *L. major* promastigotes. After the treatment of *L. infantum* promastigotes with TPF1 and TPF2 at their IC_50_ concentrations for 72 h, the number of viable parasites notably decreased from 97.8% to 53.5% and 39.4%, respectively (Figure 5A). Likewise, the treatment of *L. major* parasites with TPF1 and TPF2 at IC_50_ concentrations for 72 h, led to a decrease in viable parasites from 96.8% to 49.4% and 24.8% (*p* < 0.05) (Figure 5B). Moreover, there are statistically significant differences in the percentage of annexin V+ parasites between the groups treated with TPF1 and TPF2 and the reference drug HePC compared to the untreated control group. After treatment with TPF1 and TPF2, the percentage of the annexin V+ population increased from 1.4% to 18.9% and 22.7%, compared to the untreated *L. infantum* parasites (*p* = 0.021). The relative percentages in *L. major*-treated parasites increased from 2.7% to 23.6% and 34.5% (*p* = 0.034). Furthermore, our results showed that the treatment of *L. infantum* and *L. major* promastigotes with TPF1 and TPF2 provoked an equal increase in annexin V+ parasites as the HePC reference drug (*p* ≥ 0.05), while only TPF2-treated *L. major* promastigotes demonstrated an increased proportion of the annexin V+ population compared to HePC (*p* = 0.021). Representative dot plots with respective quadrants that illustrate early apoptotic, late apoptotic and necrotic parasites are presented in Figure 6. Triton X-100 was used as the experimental positive control of necrosis (annexin V-/PI+). Treatment with triton X-100 drove 45.9% and 49.3% of *L. infantum* and *L. major* promastigotes to suffer a loss of membrane integrity (data not shown).

### 2.7. TPF1 and TPF2 Induce DNA Fragmentation 

**Figure 7:**
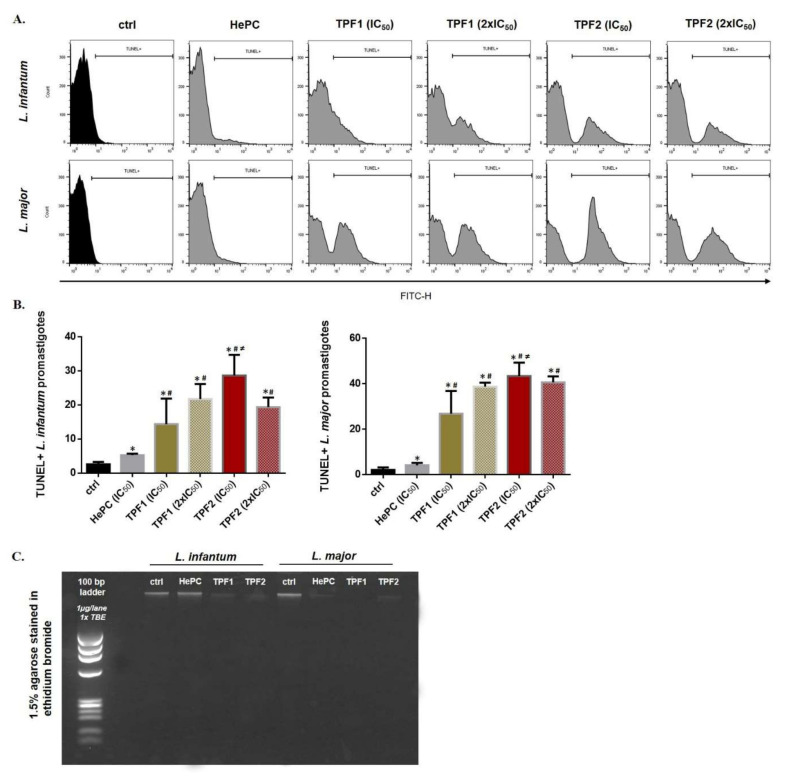
TPF1 and TPF2 induced DNA fragmentation in *L. infantum* and *L. major* promastigotes. Log-phase *L. infantum* and *L. major* promastigotes were either left untreated (negative control) or were treated with IC_50_ and 2 × IC_50_ doses of TPF1, TPF2 and HePC (positive control) for 72 h. (**A**) Flow cytometric single parameter histograms (FL1-H) representative of one experiment. (**B**) Bar diagrams illustrating the mean TUNEL+ *L. infantum* and *L. major* parasites (± SD) and are representative of three independent experiments. The symbols of * and # indicate significant differences compared to the negative and positive control groups, respectively. Significant differences between TPF1- and TPF2-treated parasites are indicated with ≠. (**C**) Genomic DNA fragmentation assay on a 1.5% agarose gel.

DNA fragmentation of log-phase *L. infantum* and *L. major* promastigotes treated with TPF1 and TPF2 for 72 h was determined by a TUNEL assay. TUNEL staining revealed a significant shift of fluorescence in the right direction in TPF1- and TPF2-treated parasites (Figure 7A). TUNEL+ *L. infantum* promastigotes were 5.5- and 11-fold increased after treatment with TPF1 and TPF2 at their IC_50_ concentration for 72 h (*p* = 0.034 and 0.021, respectively) (Figure 7B). Similarly, TUNEL+ *L. major* promastigotes were 7.8- and 12.6-fold increased after treatment with TPF1 and TPF2 at their IC_50_ concentrations for 72 h, compared to the untreated parasites (*p* = 0.021 and 0.034, respectively) (Figure 7B). We further validated the findings of TUNEL assay by performing a DNA fragmentation assay using agarose gel electrophoresis. Both TPF1 and TPF2 at their IC_50_ concentrations were able to induce marked genomic DNA fragmentation of *L. infantum* and *L. major* parasites as observed on an agarose gel (Figure 7C).

### 2.8. Anti-Leishmanial Interaction of TPF1 and TPF2 with Miltefosine

The anti-leishmanial effects of TPF1 and TPF2 in combination with HePC were investigated on promastigote and amastigote forms of *L. infantum* and *L. major*. This experimental approach allowed the determination of fractional inhibitory concentrations (FICs) for each combination. Table 3 and Table 4 show the IC_50_ values for each drug alone and in combination with HePC, as well as the FIC values and the fractional inhibitory concentration index (FICI) [31,32]. Thus, the drug combinations on promastigote forms reduced the IC_50_ values of HePC approximately up to seven times. Interactions of TPF1 and TPF2 with HePC were classified as additive with a FIC index of 0.62 and 0.81 for TPF1-HePC-treated *L. infantum* and *L. major* promastigotes, respectively (Table 3). Similarly, FIC indexes in TPF2-HePC interactions were 0.63 and 0.65 for *L. infantum* and *L. major* promastigotes, respectively (Table 4). 

**Figure 8:**
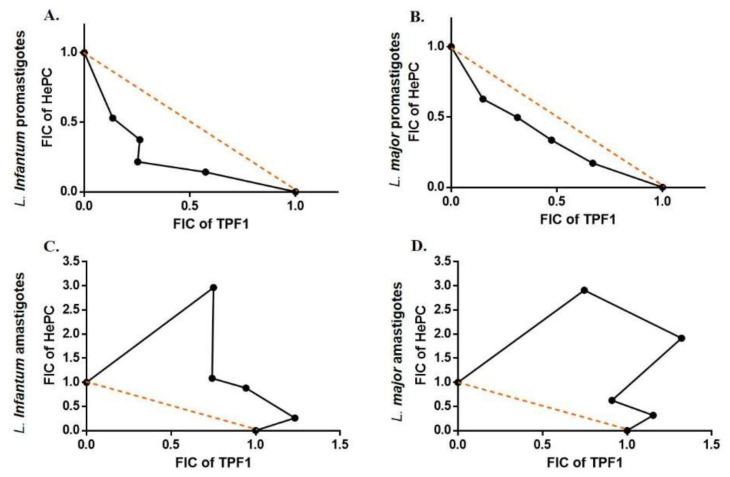
Representative normalized isobolograms of the interaction of TPF1 and HePC. Drug interaction was analyzed against *L. infantum* (**A**,**C**) and *L. major* (**B**,**D**) promastigotes and intracellular amastigotes. The numbers on the axes represent normalized FICs of TPF1 (*x*-axis) and HePC (*y*-axis). Data points (dots) located above, on, or below the line indicate antagonism, additivity, or synergy, respectively. The constructed isobolograms are the result of three independent experiments run in triplicate.

**Figure 9:**
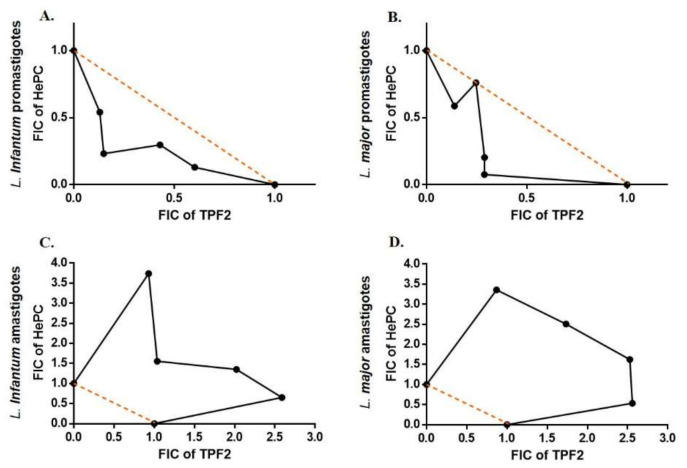
Representative normalized isobolograms of the interaction of TPF2 and HePC. Drug interaction was analyzed against *L. infantum* (**A**,**C**) and *L. major* (**B**,**D**) promastigotes and intracellular amastigotes. Numbers on the axes represent normalized FICs of TPF2 (*x*-axis) and HePC (*y*-axis). Data points (dots) located above, on, or below the line indicate antagonism, additivity, or synergy, respectively. The constructed isobolograms are the result of three independent experiments run in triplicate.

In addition, the FICI values for the TPF1-HePC combination against intracellular *L. infantum* and *L. major* amastigotes were 2.22 and 2.48, respectively (Table 3), while TPF2-HePC combinations exhibited FICI values at 3.47 and 3.93, respectively. These FICI values indicated an antagonistic interaction between each TPF and HePC against intracellular amastigotes [31,32] (Table 3 and Table 4). Lastly, the resulting effects of drug association were graphically evaluated by plotting the FIC values of the compounds in combination as isobolograms (Figure 8 and Figure 9). The points corresponding to the FIC values were connected by tendency lines, while the dashed lines representing the additivity line range of confidence were constructed by the intersection of single medication IC_50_ values.

### 2.9. Effect of Drug Interaction with Miltefosine on the Induction of Oxidative Stress in Leishmania spp. Promastigotes

**Figure 10:**
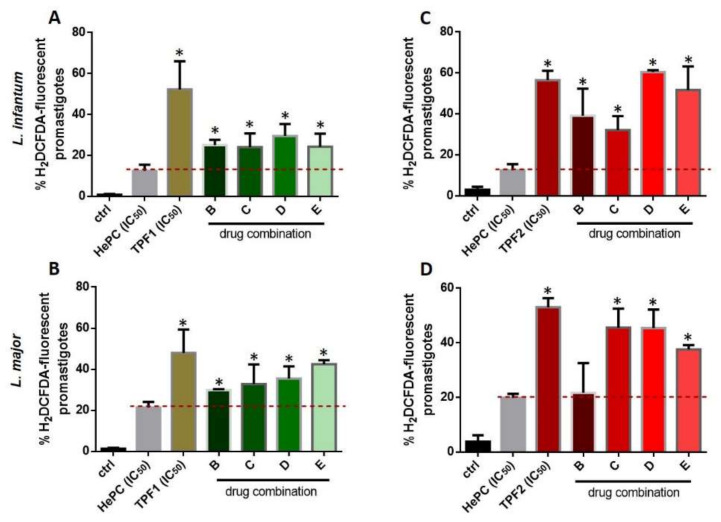
Intracellular ROS levels in *L. infantum* and *L. major* promastigotes treated with combinations of TPF1 and TPF2 with HePC. Log-phase promastigotes were treated with proportions of 4:1, 3:2, 2:3, and 1:4 of HePC and TPF1 (**A**,**B**) or TPF2 (**C**,**D**), respectively, for 72 h. Untreated parasites were used as a negative control group. ROS levels were quantified using H_2_DCFDA and flow cytometry. Data are presented as mean values of H_2_DCFDA-fluorescent promastigotes (± SD) in bar diagrams. The symbol of * indicates significant differences compared to HePC-treated parasites.

Furthermore, we tried to evaluate ROS production in drug combination-treated promastigotes compared to HePC (Figure 10). Treatment with the combination doses showed a prompt increase in ROS production, for both *L. infantum* and *L. major* promastigotes, compared to HePC. These findings confirmed the additive association between each TPF and HePC, previously determined by the isobologram analysis.

## 3. Discussion

The orientation of scientific interest in bioactive compounds of natural origin has intensified and played an important role in the development of new drugs [33,34]. During recent years, many research groups have sought to utilize natural products as effective treatments against leishmaniasis [11]. We have recently reported that a TPF containing HT, T, OLEA, and OLEO at 30, 22, 110, and 70 mg/g of extract, respectively, exhibited inhibitory effects against cell-free promastigotes and intracellular amastigotes of *Leishmania* spp. by inducing an apoptotic-like cell death [28]. Taking into consideration that the chemical characteristics of olive oils are influenced by several factors, such as olive variety, cultivation practice, the period of harvesting, weather conditions, and milling procedures, the phenolic composition and quantities among the extracted TPFs can be highly diverse [20,35]. Herein, we performed a comparative analysis of the anti-leishmanial properties of two TPFs originating from olive tree varieties grown in two different geographical areas of Greece (Peloponnese—Arcadia and Crete—Herakleion), with completely different compositions with respect to the two main chemical classes of polyphenols, i.e., phenyl alcohols (HT, and T) and secoiridoids (OLEA, and OLEO). We verified that both TPFs, despite their different phenolic composition, exerted a strong anti-leishmanial activity. The quantitative analysis revealed that TPF1 contained HT and T at 7 and 42 mg/g of extract, respectively, while TPF2 was richer in phenolic compounds as it contained HT (5 mg/g), T (12 mg/g), OLEA (144 mg/g), and OLEO (301 mg/g). The present study reported that both TPF1 and TPF2 exhibited inhibitory effects against both the promastigote and intracellular amastigote forms of *L. infantum* and *L. major*, the causative agents of VL and CL around the Mediterranean basin, while having a low toxicity against macrophages, the hostile environment of *Leishmania* spp. The SI values were higher than one, indicating a safety index for the use of these fractions in the treatment of *Leishmania*-infected host cells. In addition, our results indicated that TPF2 exhibited more potent anti-leishmanial properties compared to TPF1, as its IC_50_ values against *L infantum* and *L. major* promastigotes were 3.7- and 3.9-fold decreased. The respective IC_50_ value activity against *L infantum* and *L. major* amastigotes were two-fold decreased compared to TPF1 and the observed differences could be ascribed to their different phenolic composition. In addition, promastigote morphological changes and a decrease in growth kinetics confirmed their effect on direct parasite killing. Untreated promastigotes had the typical elongated parasitic shape, while parasites treated for 72 h with TPF1 or TPF2 presented morphological alterations, such as body deformation.

We further elucidated their comparative effectiveness as chemotherapeutic agents against leishmaniasis, by determining the type of cell death initiated in TPF1- and TPF2-treated *Leishmania* spp. promastigotes. To assess the induced type of cell death, we evaluated recently unified criteria that have been proposed for the definition of apoptosis in *Leishmania* spp. [36]. According to these criteria, after the demonstration of promastigote viability loss, we evaluated the loss of plasma membrane integrity by PI staining. Moreover, we defined apoptosis by showing the presence of at least two apoptotic markers among DNA fragmentation, cell rounding, cell shrinkage, plasma membrane modifications, and mitochondrial depolarization. Plasma membrane modifications and DNA fragmentation were evaluated by Annexin V staining and TUNEL assay, respectively, and these data also demonstrate TPF2 as the most promising against both *Leishmania* spp. Moreover, our data suggested that the promastigote death induced by TPF phenolic extracts was attributable to ROS generation. Since the loss of mitochondrial integrity for this parasite form is associated with ROS production, we suggest that our fractions could act through this process. This is also validated by our previous findings, which demonstrated that another TPF with a different phenolic composition disrupted the mitochondrial membrane potential of *Leishmania* spp. promastigotes and provoked the generation of intracellular ROS [28]. These findings are consistent with other studies which also demonstrated the contribution of ROS production and mitochondrial dysfunction to *Leishmania* death induced by natural products, such as sesamol, carajurin, apigenin, and quercetin [37,38,39,40]. 

Severe drawbacks of existing anti-leishmanial drugs, such as toxicity and parasite resistance, may be overcome with the alternative strategy of combination therapy [41,42]. In this direction, we further appraised the in vitro combination effects of TPF1 and TPF2 with miltefosine, in order to determine whether TPF1 or TPF2 was able to improve miltefosine monotherapy against *Leishmania* parasites. Four combinations of miltefosine with each one of the phenolic extracts (4:1, 3:2, 2:3 and 1:4) were investigated for their efficacy against *L. infantum* and *L. major* extracellular promastigote and intracellular amastigote forms. The analysis of the overall mean of ƩFIC, which varied from 0.469 to 0.842 for miltefosine:TPF1 combinations and 0.365 to 1.007 for miltefosine:TPF2, revealed additive effects in both promastigote *Leishmania* strains. Particularly, the analysis of the isobologram showed that the combination 2:3 (miltefosine:TPF1) was the closest to a synergistic effect against *L. infantum*, compared to the other tested combinations, reducing the required miltefosine dose by approximately five times. The respective analysis of miltefosine:TPF2 interactions showed that the 3:2 and 1:4 combinations were the closest to a synergistic effect against *L. infantum* and *L. major*, respectively. These combinations resulted in a 4.4- and 13-fold reduction of the required miltefosine dose against *L. infantum* and *L. major* promastigotes, respectively. The above advocates the more potent anti-leishmanial properties of TPF2 in comparison to TPF1. To date, few studies have reported the association between synthetic drugs and natural compounds in the battle against leishmaniasis [43,44,45]. The additive effects of each TPF combination with miltefosine according to the FIC index, were further highlighted by the enhanced intracellular ROS production in *Leishmania* promastigotes treated with drug combinations compared to miltefosine-treated parasites. Taking into account these encouraging results on extracellular promastigotes, drug interactions were also tested in intracellular amastigotes. According to the adopted classification, the results obtained in intracellular amastigotes showed that the combinations of TPF1 and TPF2 with miltefosine were considered antagonistic because the overall ƩFIC averages varied from 2.22 to 3.93. Nevertheless, the analysis of the isobologram showed that the 1:4 miltefosine:TPF1 combination was considered to be indifferent against both *L. infantum* and *L. major*, compared to the other combinations (ƩFIC values 1.493 and 1.472, respectively). 

Overall, this is the first study that demonstrates a comparative evaluation of the anti-leishmanial properties of two different TPFs derived from EVOOs, highlighting the positive impact of the presence of OLEA and OLEO. At this point, it must be highlighted that OLEA and OLEO comprise derivatives (precursors) of HT and T, respectively, and could facilitate as prodrugs releasing HT and T in a biological system after metabolization (hydrolysis). These findings are consistent with one of our previous studies, which demonstrated the remarkable in vitro and in vivo anti-leishmanial properties of pure OLEO [26]. Moreover, this is the first study that describes the nature of interactions between the phenolic extracts of EVOO and miltefosine against both a viscerotropic and a dermotropic *Leishmania* strain. Although in vitro interaction studies are promising approaches for screening drug combinations, future in vivo experiments should be conducted to assess possible drug interactions. Overall, our data suggest a need for further exploitation of bioactive compounds derived from EVOO, in order to produce strong scientific evidence of their potential additive and synergistic effects at improved therapeutic applications against leishmaniasis.

## 4. Materials and Methods

### 4.1. Starting Material

In the context of our continuation study on EVOO [20,46], two samples were selected based on their different chemical compositions regarding HT, T, OLEA, and OLEO. More specifically, EVOO-1 and EVOO-2 of the Koroneiki variety were collected during the harvesting period from 2020–2021, from two different geographical areas of Greece (Arcadia, Peloponnese, Greece and Heraklion, Crete, Greece, respectively), and were produced from two-phase olive mills. Immediately upon arrival, they were subjected to centrifugation to avoid sediment accumulation that results in rapid polyphenols decomposition. Then, samples were stored in dark, glassy vials at room temperature, and in nitrogen conditions to keep the matrixes stable. 

### 4.2. Extraction of TPFs from EVOO

TPFs were extracted from EVOO-1 and EVOO-2 raw materials according to the method proposed by the international olive council (IOC, Madrid, Spain), with some modifications. Specifically, in a 100 mL test tube, 20 g of EVOO and 50 mL of methanol/H_2_O (80:20, *v/v*) were mixed and vortexed for 120 s. After this, the mixture was placed in an ultrasonic bath for 15 min at room temperature, and then centrifuged for 25 min. The hydroalcoholic phase was evaporated and an aliquot of the dried extract (TPF) was dissolved in methanol/H_2_O (80:20, *v/v*), filtered and forwarded to HPLC-DAD analysis.

### 4.3. HPLC-DAD Analysis and Quantification 

Olive oil biophenols were determined in TPF1 and TPF2 by applying the IOC proposed analytical method, performed according to analytical conditions referred to as the IOC/T.20/Doc No 29 method (IOC, 2009) [47]. Specifically, the separation was achieved on a reversed-phase Spherisorb Discovery HS C18 column (250 × 4.6 mm, 5 µm; Supelco) using a mobile phase consisting of 0.2% aqueous orthophosphoric acid (A) and methanol/acetonitrile (50:50 *v/v*) (B), at a flow rate of 1.0 mL/min and at an ambient temperature. The injection volume was held constant at 20 µL. The applied gradient elution was as follows: 0 min, 96% A and 4% B; 40 min, 50% A and 50% B; 45 min, 40% A and 60% B; 60 min, 0% A and 100% B; 70 min, 0% A and 100% B; 72 min, 96% A and 4% B; and 82 min, 96% A and 4% B. Chromatograms were monitored at 280 nm. All analyses were performed in triplicate.

Concentration levels of major biophenols were determined using a regression analysis method. Specifically, standard calibration curves of hydroxytyrosol (HT), tyrosol (T), oleacein (OLEA), and oleocanthal (OLEO) were prepared. For the HT and T quantification, 9-point calibration curves were constructed (HT: y=84028x+39609, r^2^ = 0.9997; T: y=53933x−11712, r^2^ = 0.9987), while OLEA and OLEO were quantified according to their 8- and 10-point calibration curves, respectively (OLEA: y=32720x+12723, r^2^ = 0.9997 and OLEO: y=18836x+54185, r^2^ = 0.9982). The results were expressed in mg analyte per g of TPF. HT and T reference standards were purchased from ExtraSynthase (Lyon Nord, France) while OLEO and OLEA were purchased from Pharmagnose SA (Oinofyta, Greece). The selected TPFs were dissolved in dimethyl sulfoxide (DMSO, Applichem, Germany) and further diluted in cell culture medium in assays. The final concentration of DMSO was less than 0.1% in all in vitro assays.

### 4.4. Parasite and Eukaryotic Cell Culture

*L. infantum* (zymodeme GH8, strain MHOM/GR/2001/GH8) and *L. major* (zymodeme LV39, strain MRHO/SU/59/P) parasites, causative agents of visceral and cutaneous leishmaniasis, respectively, were used in this study. Promastigotes were grown at 26 °C in complete RPMI-1640 medium (PAN-Biotech, Aidenbach, Passau, Germany) in cell culture flasks (SPL Life Sciences, Naechon-Myeon, Pocheon-si, Korea), as previously reported [48].

The immortalized macrophage cell line J774A.1 (ATCC No: TIB-67) was cultured in 25 cm^2^ cell culture flasks (ThermoFisher Scientific, Waltham, MA, USA), at 37 °C with a 5% CO_2_ environment [48]. 

### 4.5. In Vitro Activities against L. infantum and L. major Promastigotes

The anti-leishmanial activities of TPFs, were evaluated against log-phase *L. infantum* and *L. major* promastigotes using the resazurin cell viability assay. Briefly, promastigotes were prepared in RPMI-1640 medium supplemented with 10% fetal bovine serum (FBS) and the promastigote suspensions were plated in 96-well flat-bottom cell culture plates (2 × 10^6^ *L. infantum* and 2.5 × 10^6^ *L. major* per well) at a final volume of 200 µL. Promastigotes were exposed to different increasing concentrations of TPFs (800–1500 µg/mL and 100–850 µg/mL for TPF1 and TPF2, respectively), in triplicate. Promastigotes exposed only in culture medium were considered to be the negative control group, while parasites exposed to the reference drug of miltefosine (HePC, Virbac) were considered to be the positive control group (2.48 µg/mL for *L. infantum* and 3.38 µg/mL for *L. major*). Plates were incubated at 26 °C for 65 h. After exposure to the drug, resazurin solution (20 µg/mL) was added to each well and the plates were further incubated for 24 h at 26 °C. The promastigote proliferation was determined using a microplate spectrophotometer at 570 nm (reference filter at 630 nm). The percentage (%) of viable parasites was calculated by using the mean of the negative control as 100% survival of the parasite. Data were normalized using the formula: % of survival = (sample OD − blank OD)/(control OD − blank OD) × 100. The half-maximal inhibitory concentration (IC_50_) values were determined from a dose-response curve generated using Excel 2016 software, as previously described [27,49]. Three independent experiments with triplicates within each experiment, were performed. The results are expressed as mean ± SD.

### 4.6. In Vitro Cytotoxicity Assay

J774A.1 macrophages were plated at 4 × 10^4^ cells/well in 96-well flat bottom plates [27,49] and treated with different concentrations of each TPF (150–350 µg/mL and 60–220 µg/mL for TPF1 and TPF2, respectively). After 72 h, cell viability was measured by a resazurin assay by adding resazurin at 20 μg/mL. HePC was used as a positive control. The CC_50_ value is expressed as the cytotoxic concentration for 50% of cells. Data were normalized using the aforementioned formula. Three independent experiments with triplicates within each experiment, were performed. The results are expressed as mean ± SD. 

### 4.7. In Vitro Activities against L. infantum and L. major Amastigotes

Anti-amastigote killing activity within J774A.1 macrophages was assessed with a resazurin cell viability assay. Macrophages at 5 × 10^4^ cells/well were plated in 96-well flat bottom plates in RPMI-1640 medium supplemented with 10% FBS. The cells were incubated at 37 °C under 5% CO_2_ humidified air for 18 h in order to achieve cell adhesion [49]. After this, the cells were infected with log-phase promastigotes at a 1:15 ratio for 48 h. Non-internalized promastigotes were washed with RPMI-1640 medium and the tested compounds were added in increasing concentrations (80–300 µg/mL for TPF1 and 20–200 µg/mL for TPF2). HePC was used as a reference drug. After 48 h of incubation, the supernatant was removed and the cell membranes were disrupted. The culture medium was replaced by 200 µL/well of complete Schneider’s insect medium and the plates were further incubated at 26 °C for 72 h in order to achieve the transformation of viable amastigotes into promastigotes. Finally, 5 µL of resazurin solution (60 µg/mL) was added to each well and the plates were further incubated for 24 h at 26 °C. The amastigote proliferation was determined using a microplate spectrophotometer. The obtained values of each concentration were used to obtain the IC_50_ value for the intracellular amastigotes [49]. Three independent experiments with triplicates within each experiment, were performed. The results are expressed as mean ± SD.

### 4.8. Effects of TPF1 and TPF2 on Growth Kinetics of L. infantum and L. major Promastigote Cultures

*L. infantum* and *L. major* log-phase promastigotes were treated with TPF1 and TPF2 at IC_50_ and 2 × IC_50_ concentrations. Promastigotes cultured only under the presence of culture medium represented the negative control group, while HePC (IC_50_)-treated parasites were the positive control group. Parasite growth and multiplication were monitored by differential counting of dead and live promastigotes at 24 h intervals for 3 consecutive days with the use of Trypan blue exclusion dye (Sigma–Aldrich, St. Louis, Mo, USA) in a Malassez counting chamber (hemocytometer) under a binocular optical microscope (Olympus, Shinjuku-ku, Tokyo, Japan), as previously described [28]. The morphology of the promastigotes was observed under a phase-contrast microscope with a 40x objective (Leica DMi1 inverted microscope).

### 4.9. Detection of Reactive Oxygen Species (ROS) Production in L. infantum and L. major Promastigotes

ROS production was detected using cell permeable H_2_DCFDA (2′,7′-dichlorodihydro-fluorescein diacetate, Life Technologies, NY, USA), as previously described [28,50]. *L. infantum* and *L. major* log-phase promastigotes were treated with TPF1 and TPF2, respectively, for 72 h at 26 °C, at IC_50_ and 2 × IC_50_ concentrations. In the experiments of drug combination, the parasites were treated with various proportions of HePC:TPF1 and TPF2 independently (4:1, 3:2, 2:3, 1:4), as described below in Section 4.13. Then, 5 × 10^6^ parasites/group were centrifuged, washed twice with phosphate buffered saline (PBS), and incubated with 20 µM H_2_DCFDA for 20 min in the dark, at room temperature. Hydrogen peroxide (1 mM) was used as a positive control. Fluorescence intensity was measured by flow cytometry. Samples (20,000 promastigotes/group) were assessed on FACSCalibur (BD, San Jose, CA, USA) and the acquired data were analyzed with FlowJo V.10.0.8 software (BD, Franklin Lakes, NJ, USA).

### 4.10. Annexin V Binding and Propidium Iodide Staining

*L. infantum* and *L. major* log-phase promastigotes were treated with TPF1 and TPF2, respectively, for 72 h at 26 °C, at IC_50_ and 2 × IC_50_ concentrations. Promastigotes treated with HePC (IC_50_) were considered as the positive control group, while untreated parasites were the negative control group. Triton X-100 (1% *v/v*) was used as a control for 100% membrane permeabilization. Parasites were labeled with annexin V and propidium iodide (PI) using the annexin V-fluorescein isothiocyanate (FITC) apoptosis detection kit (BioLegend, San Diego, CA, USA), according to the manufacturer’s instructions. Briefly, 10^6^ parasites/group were centrifuged at 1600 rpm for 10 min at room temperature, washed twice in PBS and resuspended in 100 µL of annexin V binding buffer, 5 µL of annexin V-FITC, and 10 µL of PI solution. Parasites were incubated for 15 min at room temperature, protected from the light. At the end of the incubation period, 400 µL of annexin V binding buffer was added in each sample and the samples were analyzed by flow cytometry. The samples (20,000 promastigotes/group) were assessed on FACSCalibur and the acquired data were analyzed with FlowJo V.10.0.8 software. Promastigotes were classified according to their staining as early apoptotic (annexin V+/PI-), late apoptotic (annexin V+/PI+), necrotic (annexin V-/PI+), and viable parasites (annexin V-/PI-).

### 4.11. Terminal Deoxyribonucleotidyl Transferase (TdT)-Mediated dUTP Nick-End Labelling (TUNEL) Assay

A TUNEL assay was performed according to the manufacturer’s instructions (CFTM 488A TUNEL Assay, Biotium, Fremont, CA, USA). Briefly, *L. infantum* and *L. major* log-phase promastigotes were treated with TPF1 and TPF2, respectively, for 72 h at 26 °C, at IC_50_ and 2 × IC_50_ concentrations. Promastigotes treated with HePC (IC_50_) were considered as the positive control group. A total of 2 × 10^6^ parasites per experimental group, were harvested by centrifugation (1600 rpm, 10 min) and washed twice with 1 mL of PBS. The parasites were then fixed by adding 4% formaldehyde/PBS and incubated at 4 °C for 30 min. The parasites were resuspended in permeabilization buffer containing 0.2% Triton X-100 and incubated for 30 min at room temperature. The cells were washed twice in PBS and then were resuspended in 100 µL of equilibration buffer and incubated at room temperature for 5 min. Equilibration buffer was removed and the cells were resuspended in 50 µL of TdT reaction mix and incubated at 37 °C for 60 min, protected from direct light. Then, the cells were washed thrice in PBS containing 0.1% Triton X-100 and 5 mg/mL BSA and analyzed on FACSCalibur.

### 4.12. DNA Extraction Protocol 

The parasites (5 × 10^6^ parasites/mL) were obtained from all experimental groups after 72 h of treatment and were lysed in 200 µL of lysis buffer supplemented with proteinase K and allowed to digest at 56 °C for 10 min. Total genomic DNA was obtained using a DNA Mini Kit (Qiagen, Hilden, Germany), according to manufacturer’s instructions. The DNA concentration and purity were determined by measuring optical density (OD) on a NanoDrop 2000 spectrophotometer (Thermo Scientific, Waltham, MA, USA). Total genomic DNA was separated on a 1.5% agarose gel containing ethidium bromide. The gel was run for 50 min at 120 V and visualized by an Imaging System (Bio-Rad, Hercules, CA, USA).

### 4.13. Assessment of Drug Interactions 

The modified isobologram method was used in order to determine the interaction between each TPF with HePC [43,51]. The drug interactions were assessed in both *L. infantum* and *L. major* log-phase promastigotes, as well as in intracellular amastigotes. The IC_50_ value of each compound (TPF1, TPF2, HePC), previously determined, was used to calculate the top concentration of each drug to ensure that each IC_50_ value was close to the middle of a six-point twofold dilution series. The top concentrations were prepared in four proportions of HepC with each TPF. The tested associations were as follows: 4:1 (combination B = 80% HePC + 20% TPF), 3:2 (combination C = 60% HePC + 40% TPF), 2:3 (combination D = 40% HePC + 60% TPF), and 1:4 (combination E = 20% HePC + 80% TPF). Experiments were carried out using the same approach for the evaluation of anti-leishmanial activity, using the resazurin viability assay as described in Section 4.5 and Section 4.7. Thus, after 72 h of incubation, an IC_50_ value for each drug in combination was obtained for each ratio.

### 4.14. Determination of Fractional Inhibitory Concentrations (FICs) Index 

The fractional inhibitory concentrations (FICs) at the IC_50_ level for each drug ratio (4:1, 3:2, 2:3 and 1:4) were calculated based on the following equation: FIC = IC_50_ of each drug in combination/IC_50_ of each drug alone. The isobologram construction association curves were determined by using the FICs of each drug ratio. Afterwards, the sum of FICs for each ratio was calculated by the formula ƩFIC = FIC_TPF_ + FIC_HePC_ and finally, as an overall mean of ΣFICs, the fractional inhibitory concentration index (FICI) was determined. The obtained FICI values were used to classify the nature of the interaction as: synergistic (FICI ≤ 0.5), additive (0.5 < FICI ≤ 1, indifferent (1 < FICI < 2), and antagonistic (FICI ≥ 2) [31,32].

### 4.15. Statistical Analysis

Data are representative of at least three independent experiments and are presented as mean values ± SD. The differences between the means were analyzed for significance using the two-sided Mann–Whitney test (IBM SPSS Statistics for Windows, Version 23.0. Armonk, NY, USA: IBM Corp.) and were considered significant at a 0.05 level of confidence.

## Figures and Tables

**Table 1:** Phenolic composition of the analyzed TPF1 and TPF2 extracts. Data of the regression model (linear regression, r-squared and concentration ranges) are given.

Phenolic Compounds	TPF1	TPF2	Linearity of Phenolic Compounds Standards		
	mg/g of Extract (Mean ± SD, n = 3)		Linear Regression	r^2^	Concentration Range (µg/mL)
HT	7.02 (±0.4)	5.01 (±0.2)	y=84028x+39609	0.9997	0.5–100
T	42.07 (±0.2)	12.03 (±0.1)	y=53933x−11712	0.9987	0.5–100
OLEA	Non determined	144.12 (±5.4)	y=32720x+12723	0.9997	5–500
OLEO	Non determined	301.24 (±6.5)	y=18836x+54185	0.9982	5–600

**Table 2:** Cytotoxicity evaluation and anti-leishmanial activity of individual drugs against *Leishmania infantum* and *Leishmania major* promastigotes and intracellular amastigotes.

Compound	Cytotoxicity (J774A.1 Cells)	*L. infantum*			*L. major*		
		Promastigotes	Amastigotes		Promastigotes	Amastigotes	
	CC_50_ (µg/mL)	IC_50_ ± SD (µg/mL)		SI	IC_50_ ± SD (µg/mL)		SI
TPF1	270.22 ± 8.14	1186.48 ± 45.82	207.02 ± 6.57	1.3	976.03 ± 21.56	142.3 ± 28.24	1.9
TPF2	157.6 ± 2.18	322.58 ± 18.87	104.98 ± 10.02	1.5	252.58 ± 30.16	76.07 ± 14.5	2.07
HePC	28.48 ± 3.66	2.48 ± 0.23	1.57 ± 0.84	18.14	3.38 ± 0.3	2.36 ± 0.17	12.06

Data represent mean ± SD of three independent experiments performed in triplicate. CC_50_: cytotoxic concentration for 50% of cells; IC_50_: inhibitory concentration for 50% of parasites; SI (selectivity index) = CC_50_ for J774A.1 cells/IC_50_ for amastigotes.

**Table 3:** IC_50s_, FICs and ∑FICs of TPF1 and HePC combinations against *Leishmania infantum* and *Leishmania major* promastigotes and intracellular amastigotes.

Parasite Form	Combined Drugs									Nature of Interaction
	Combination	Concentrations (µg/mL)		IC_50_ ± SD (µg/mL)		FIC		∑FIC	FICI	
		HePC	TPF1	HePC	TPF1	HePC	TPF1			
Promastigotes *L. infantum*	A	20	0	2.48 ± 0.23	-	-	-	-		
	B	16	1760	1.31 ± 0.31	160.74 ± 1.46	0.530	0.135	0.665		
	C	12	3520	0.93 ± 0.29	312.12 ± 24.21	0.374	0.263	0.637	0.62	Additive
	D	8	5280	0.54 ± 0.17	299.95 ± 15.83	0.216	0.253	0.469		
	E	4	7040	0.35 ± 0.07	681.03 ± 39.12	0.142	0.574	0.716		
	F	0	8800	-	1186.48 ± 45.82	-	-	-		
Promastigotes *L. major*	A	28	0	3.38 ± 0.3	-	-	-	-		
	B	22.4	1540	2.12 ± 0.04	145.95 ± 2.43	0.628	0.150	0.777		
	C	16.8	3080	1.68 ± 0.03	306.58 ± 5.14	0.497	0.314	0.811	0.81	Additive
	D	11.2	4620	1.13 ± 0.02	463.93 ± 12.34	0.336	0.475	0.811		
	E	5.6	6160	0.58 ± 0.02	653.53 ± 34.05	0.172	0.670	0.842		
	F	0	7700	-	976.03 ± 21.56	-	-	-		
Amastigotes *L. infantum*	A	12	0	1.57 ± 0.84	-	-	-	-		
	B	9.6	320	4.66 ± 0.39	155.51 ± 13.48	2.966	0.751	3.718		
	C	7.2	640	1.70 ± 0.20	153.77 ± 20.02	1.084	0.743	1.827	2.22	Antagonistic
	D	4.8	960	1.39 ± 0.36	195.27 ± 5.85	0.883	0.943	1.826		
	E	2.4	1280	0.41 ± 0.11	254.96 ± 14.44	0.261	1.232	1.493		
	F	0	1600	-	207.02 ± 6.57	-	-	-		
Amastigotes *L. major*	A	18	0	2.36 ± 0.17	-	-	-	-		
	B	14.4	224	6.87 ± 1.33	106.36 ± 21.03	2.909	0.747	3.657		
	C	10.8	448	4.52 ± 0.97	188.11 ± 37.90	1.916	1.322	3.238	2.48	Antagonistic
	D	7.2	672	1.48 ± 0.27	129.54 ± 13.43	0.627	0.910	1.538		
	E	3.6	896	0.75 ± 0.17	164.39 ± 16.03	0.317	1.155	1.472		
	F	0	1120	-	142.3 ± 28.24	-	-	-		

FIC, fractional inhibitory concentration at the indicated IC_50_. ∑FIC, sum of the FICs. FICI, fractional inhibitory concentration index.

**Table 4:** IC_50s_, FICs and ∑FICs of TPF2 and HePC combinations against *Leishmania infantum* and *Leishmania major* promastigotes and intracellular amastigotes.

Parasite Form	Combined Drugs									Nature of Interaction
	Combination	Concentrations (µg/mL)		IC_50_ ± SD (µg/mL)		FIC		∑FIC	FICI	
		HePC	TPF2	HePC	TPF2	HePC	TPF2			
Promastigotes *L. infantum*	A	20	0	2.48 ± 0.23	-	-	-	-		
	B	16	500	1.34 ± 0.37	41.9 ± 11.61	0.540	0.130	0.669		
	C	12	1000	0.57 ± 0.02	48.16 ± 2.7	0.232	0.149	0.380	0.63	Additive
	D	8	1500	0.74 ± 0.06	135.8 ± 13.78	0.297	0.429	0.726		
	E	4	2000	0.32 ± 0.13	194.2 ± 6.12	0.130	0.602	0.732		
	F	0	2500	-	322.58 ± 18.87	-	-	-		
Promastigotes *L. major*	A	28	0	3.38 ± 0.3	-	-	-	-		
	B	22.4	400	1.98 ± 0.70	35.41 ± 12.51	0.586	0.140	0.726		
	C	16.8	800	2.57 ± 0.71	62.42 ± 19.50	0.760	0.247	1.007	0.65	Additive
	D	11.2	1200	0.69 ± 0.25	73.27 ± 27.02	0.203	0.290	0.493		
	E	5.6	1600	0.26 ± 0.18	73.02 ± 49.90	0.076	0.289	0.365		
	F	0	2000	-	252.58 ± 30.16	-	-	-		
Amastigotes *L. infantum*	A	12	0	1.57 ± 0.84	-	-	-	-		
	B	9.6	160	5.87 ± 0.87	97.66 ± 14.60	3.738	0.930	4.668		
	C	7.2	320	2.44 ± 0.29	108.85 ± 13.04	1.554	1.037	2.590	3.47	Antagonistic
	D	4.8	480	2.12 ± 0.28	212.31 ± 32.95	1.350	2.022	3.372		
	E	2.4	640	1.03 ± 0.19	271.56 ± 43.90	0.653	2.587	3.240		
	F	0	800	-	104.98 ± 10.02	-	-	-		
Amastigotes *L. major*	A	18	0	2.36 ± 0.17	-	-	-	-		
	B	14.4	120	7.93 ± 1.50	66.21 ± 12.65	3.360	0.870	4.231		
	C	10.8	240	5.92 ± 1.15	131.94 ± 4.07	2.509	1.734	4.244	3.93	Antagonistic
	D	7.2	360	3.84 ± 0.54	192.23 ± 26.83	1.626	2.527	4.153		
	E	3.6	480	1.26 ± 0.53	194.64 ± 52.07	0.536	2.559	3.095		
	F	0	600	-	76.07 ± 14.5	-	-	-		

FIC, fractional inhibitory concentration at the indicated IC_50_. ∑FIC, sum of the FICs. FICI, fractional inhibitory concentration index.

## Data Availability

Not applicable.

## Sample Availability

Samples of the compounds are available from the authors.
